# Image based Digitisation of Entomology Collections: Leveraging volunteers to increase digitization capacity

**DOI:** 10.3897/zookeys.209.3146

**Published:** 2012-07-20

**Authors:** Paul Flemons, Penny Berents

**Affiliations:** 1 Australian Museum, 6 College Street, Sydney 2010

**Keywords:** Digitising, image, volunteers, Australian Museum, collections

## Abstract

In 2010, the Australian Museum commenced a project to explore and develop ways for engaging volunteers to increase the rate of digitising natural history collections. The focus was on methods for image-based digitising of dry pinned entomology collections. With support from the Atlas of Living Australia, the Australian Museum developed a team of volunteers, training materials and processes and procedures.

Project officers were employed to coordinate the volunteer workforce. Digitising workstations were established with the aim of minimising cost whilst maximising productivity and ease of use. Database management and curation of material before digitisation, were two areas that required considerably more effort than anticipated.

Productivity of the workstations varied depending on the species group being digitised. Fragile groups took longer, and because digitising rates vary among the volunteers, the average hourly rate for digitising pinned entomological specimens (cicadas, leafhoppers, moths, beetles, flies) varied between 15 to 20 per workstation per hour, which compares with a direct data entry rate of 18 per hour from previous trials.

Four specimen workstations operated four days a week, five hours a day, by a team of over 40 volunteers. Over 5 months, 16,000 specimens and their labels were imaged and entered as short records into the museum’s collection management database.

## Introduction

The Australian Museum (AM) has natural science collections dating from 1806. The collections hold more than 18 million specimens of animals, fossils, rocks and minerals. Digitisation (in the form of databasing the text from specimen labels) of the collections commenced in the 1970s and in 2012 approximately 40% of the collections have a text record in the Museum’s collection database. To digitise the remainder of the collections in this way, would at comparable rates, take another 50 years at least.

Funding for digitising of collections needs to be allocated as efficiently and wisely as possible to maximise the return for the investment. However it is unlikely that funding available for digitising is ever going to equal funding required for fully digitising our collections. This represents the digitising impediment.

In response to a lack of adequate resources for digitising, the Australian Museum (AM) has been exploring opportunities for engaging volunteers in image-based specimen digitisation since 2007. Initial work (Tann and Flemons 2008) demonstrated that utilising volunteers for imaging specimens and their labels was feasible and compared favourably with traditional text-only data entry techniques.

In 2010 the Australian Museum obtained funding from the Atlas of Living Australia (ALA) to develop a volunteer-based digitisation program (DigiVol). The aim of this project was to explore and develop methods and technologies for engaging volunteers to assist in the rapid digitisation and registration of museum specimens. The project focused on the entomology collection, in part because it is a big collection that is largely not digitised, yet it lends itself to a methodical volunteer-based digitising process.

It was considered essential to establish a clear project scope for setting boundaries within which to develop processes and procedures. This was particularly important for the imaging process as the choice of imaging resolution would have an enormous impact on downstream use and storage of images. Computer storage costs, network bandwidth and display capabilities often lag behind the capacity for capturing high resolution images. However, the time consuming handling of specimens suggested maximising image resolution. Staying focused on the goal at hand simplified this dilemma. The primary goal in this case was to obtain good quality label images that could be easily read; the secondary goal being to capture an image of the specimen at the same time. With this in mind we established the following criteria for the project:

• Maximum 5MB file size

• Create an image of clearly readable text on labels, this being the priority

• Produce a clear, focused image of specimens at maximum resolution allowed by inclusion of labels in the same image – these specimens which will range in size from large cicada’s and moths (measured in many cm’s) to small beetles and flies (measured in the few mm’s) so detail that can be captured will vary.

• Attach a registration number

• Use relatively low cost imaging and computing equipment.

• Create a partial record of metadata, including the species name and registration number.

• Develop simple standardised processes that could be easily replicated and implemented on multiple workstations by volunteers

• Develop a process that would be comparable in speed to direct data entry by volunteers

• Ensure specimen safety with minimal breakages

• Maintain a harmonious working relationship with collection staff

This paper outlines the methodology of this project (for more detail on the materials and methods see [AM digitisation final report]), reports on the outputs, and discusses the issues encountered and lessons learned.

## Methods

### Database for storage of image metadata

An important component of digitising infrastructure was the database in which image metadata and short record information was initially entered into by the volunteers. This database was separate from the corporate collection management database for a number of reasons:

• Data Security - the corporate database had strict permissions on access for purposes of maintaining data integrity. In this project, volunteer staff, did not have data entry access

• Direct data entry into the corporate database can be slow and not as efficient or effective as using a lightweight MS Access database for data entry and validation followed by bulk importing the records into the corporate database

We chose MS Access as the platform for this database because:

• There was an existing software licence for MS Access

• The database support officer for this project has existing expertise in setting up, managing and programming in MS Access.

Where possible information stored in the database was made available as pick lists so that data entry required as little typing as possible, reducing input error and making the process faster.

**Figure 1.**
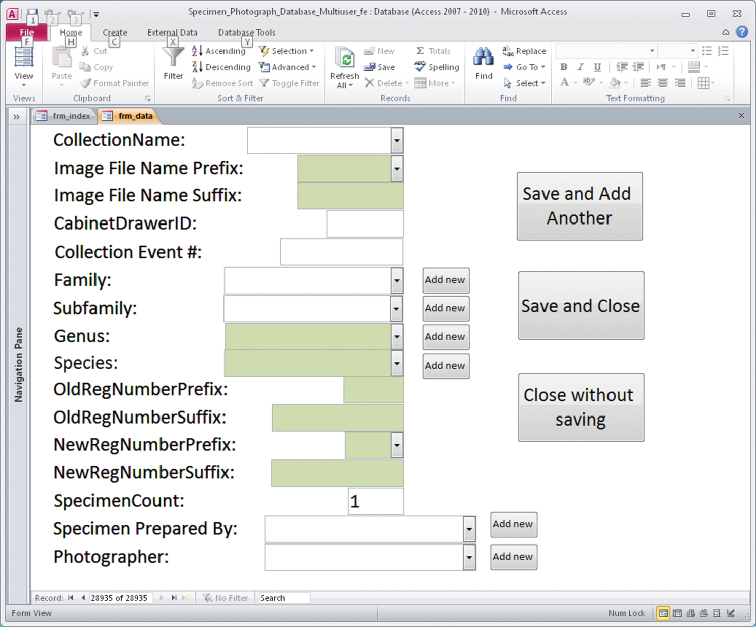
Database for entry of image metadata.

At the time of image capture volunteers enter information through the MS Access database data entry form (Figure 1).

The data captured by the volunteers includes data necessary for creating a “short record”. It contained the bare minimum of detail about a specimen to enable the creation of a valid collection database record in the museum’s collection management database, EMu. This short record which consisted of catalogue number, species (or a higher taxon level) name and the images themselves were imported into EMu. The rest of the label data, once captured could then be appended to this short record at a later date. The short record in the meantime is available for audit purposes, and for some collection and data management activities, as the specimen label data can clearly be seen on the image even though it is not text searchable.

**Figure 2.**
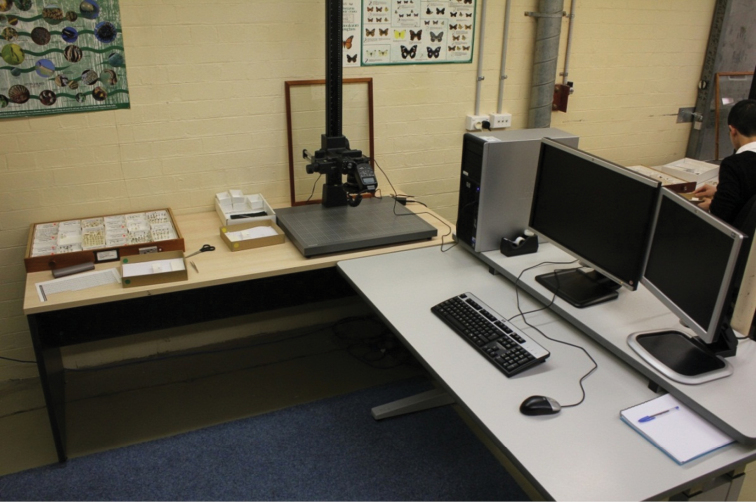
A digitising workstation.

**Figure 3.**
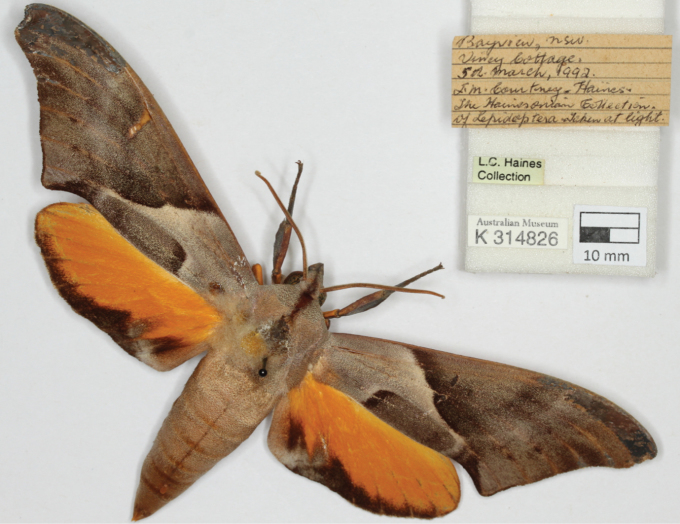
An example of a specimen and label image, in this case a hawk moth.

### Digitisation Laboratory

The Australian Museum provided a large room in which the Digitising Laboratory was established. This space was important in establishing the sense of belonging for the volunteers as it was a dedicated space for the project. The room was fitted out with power and network outlets and secure access. There was enough space for four specimen label imaging workstations, one register imaging station, a microscope camera workstation and three transcription workstations.

### Specimen workstations

Each of the four specimen imaging workstations had the same equipment. Workstations were used for imaging individual specimens and their labels.

Workstation equipment:

– Two desks

– A desktop computer capable of manipulating large images

– Camera, lens and light source (flash)

– Copy stand for vertical photography

– Tools for specimen handling such as tweezers

– For a full listing of equipment see [AM digitisation final report]

### Process/workflow

Selecting and providing specimens for digitisation involved the Museum collection staff selecting appropriate curated drawers of specimens in preparation for imaging. Selection criteria included ensuring that the taxonomy and names for specimens in drawers were as up-to-date as possible, and unambiguous. Specimens also needed to be mounted, relatively robust (this is not essential but inclusion of less robust taxa generally led to more breakages and so required more collection staff time in resolving breakages), to avoid damage when being handled, and accessible within each drawer, for example, not cramped where labels and specimens would be damaged.

It was a large task to ensure the supply of specimens for digitising. With four workstations operating four days per week, the rate at which specimens can be digitised put a considerable burden on collection staff. Curated drawers needed to be allocated for up to a month in advance to ensure that volunteers would not run out of specimens to digitise.

### Summary of the steps in handling and imaging of specimens

(for full details see [Specimen Training guide])

• Curate specimens

Before moving drawers of specimens to the imaging laboratory, collection staff ensured they were curated to a specified level for the project. Type specimens were removed, as they were considered too precious to be handled by those without appropriate training. Each specimen was checked to ensure it was labelled adequately with its taxonomic name, and that drawers were not overcrowded. This workload had significant resource implications for collection staff (see Discussion and Conclusion Table 1)

• Retrieve specimens

Drawers were removed from the collection by the digitisation officer in the order that they were numbered and transported to the Digitisation Lab. Drawers were transported on a trolley from the collection to the Digitisation Lab.

• Prepare specimens

At each workstation there were two volunteers: one volunteer handled the specimen (the specimen handler), the other volunteer photographed the specimen (the digitiser).

For more details of the process see [Specimen Training guide].

• Image specimens

Label information was entered into the database, and the specimen and its labels were imaged.

• Deal with damaged specimens/labels

Damaged specimens (broken parts are collected and included with the specimen), damaged labels, and specimens without labels were placed in a ‘hospital’ drawer to be returned to collection staff for assessment and repairs. Place holders were used to identify where the specimens were to be returned to. The collection manager was notified when the hospital drawer was full.

• Return specimen drawer to the collection

After a specimen had been imaged it was replaced in its drawer.

Once imaging of all specimens in a drawer is finished, a drawer could be returned to the collection.

• Review image and entered data

After each drawer had been imaged the information entered in the database was reviewed to ensure consistency and identify any obvious image or data capture problems.

• Monitoring of the process

The following information is recorded to monitor the project’s outputs, staffing and volunteers:

Number of specimens digitised per day by volunteer

Number of drawers digitised per day

Number of volunteer hours per day

Number of damaged specimens

Number of specimens damaged beyond repair

Number of collection staff hours per day

**Table 1.** This table gives some idea of the difficulty in estimating time required for curation. These are figures provided by David Britton, Collection Manager at AM.

Group	No. of drawers	No. of specimens	Curation time estimate
Notodontidae (moths)	26	~ 1000	7 days, included some identification (4 drawers)
Sphingidae (hawk moths)	50	1916	8–15 days, included some identification (6 drawers)
Cicadas	82	4386	25 days
Scarabaeidae (beetles)	5	2204	15 days including identification
Noctuidae (moths)	10	809	4 days
Leafhoppers etc	41	3385	20 days

### Volunteer recruitment, supervision and management

Recruitment, coordination and supervision of volunteers

The development and management of a team of well trained, productive volunteers dedicated to the digitisation of museum collections, whether large or small, required the same basic approach.

A volunteer coordinator was essential. In practice, this role and its responsibilities could have been spread across one or more people or positions. Ideally, however, we felt that a single position, which in the case of this project was shared between two individuals, was likely to produce the best outcome for the museum. The large size of the volunteer team and the high throughput of the project required a dedicated co-ordinator resource to ensure that the workload of existing staff was not impacted greatly.

The volunteer coordinators were responsible for recruiting, training, coordinating and supervising volunteers. First and foremost they needed to have excellent people management skills and a good understanding of the technical processes involved in digitisation. The extent to which they need to be technically proficient was dependent on the availability of other sources of technical expertise. Coordinators were trained in specimen handling, the extent that they assisted the collection staff in developing a video demonstrating how volunteers should handle specimens.

### The major steps in the creation of the volunteer team were as follows:

• Recruitment

An expression of interest email was sent out to Australian Museum Members. Potential volunteers were asked to identify their preferred days, which could be a Saturday, and their availability, to volunteer for one day a week, or one day a fortnight.

• Rostering

Potential volunteers were prioritised on their day preferences according to their response time to the expression of interest.

• Induction

New volunteers were given an introduction to the working area and other volunteers and a tour of the public exhibition within the museum.

• Training

Volunteers attended a one day training session with short videos about handling and imaging specimens. The videos were accompanied by training manuals, and followed by hands-on practice with experienced volunteers.

• Review

Digitiser volunteers undertook a six-week introductory period. At the end of this period each volunteer would complete a self-assessment review of their practice and the project.

• Ongoing Support

Digitiser volunteers received ongoing practise support from peers as well as the digitisation officer.

## Results

Each specimen digitised resulted in a “short” record in EMu with taxon and registration number linking with existing taxonomic information in EMu, and included an image of the specimen with associated labels.

The following graphs show various statistics over the period of the project.

**Figure 4.**
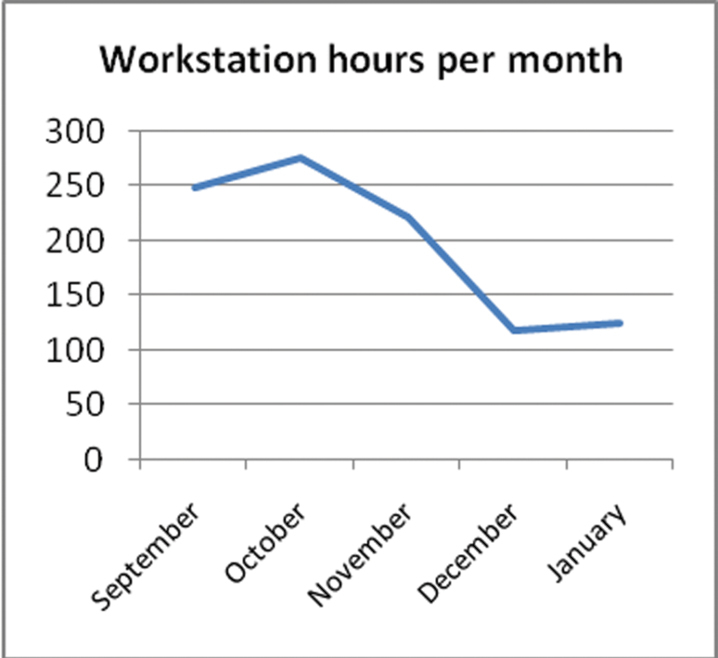
Workstation hours per month.

**Figure 5.**
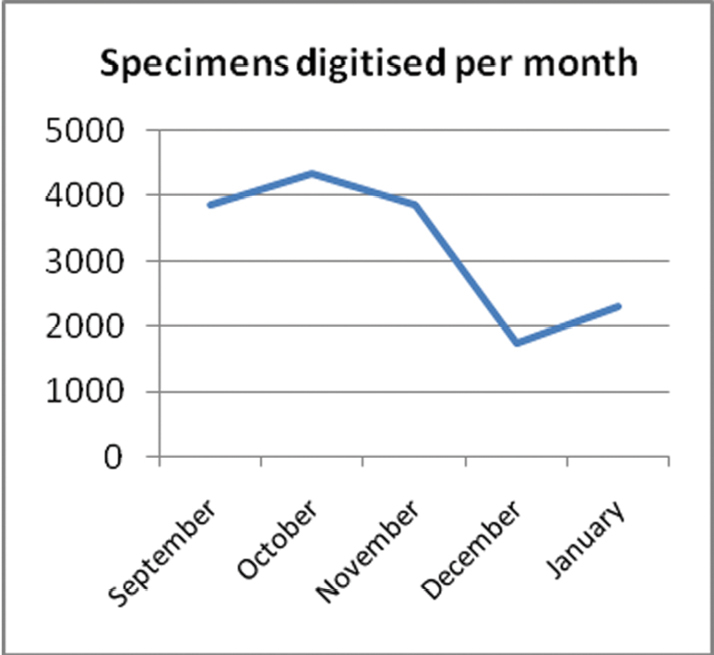
Specimens digitised per month.

As the number of workstation hours per month varied (Fig. 4) so did the number of specimens digitised per month (Fig. 5). The drop off over December was due to the Christmas holiday period.

**Figure 6.**
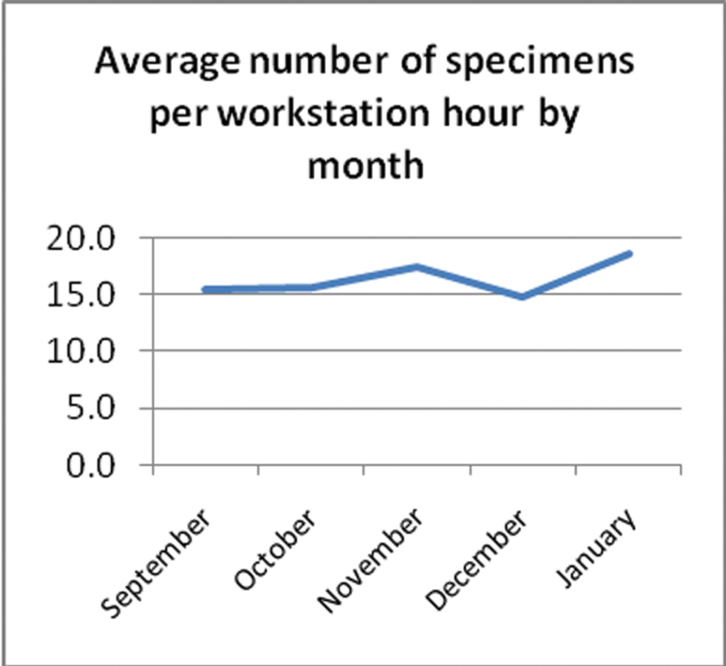
Average number of specimens per workstation hour by month.

The number of specimens digitised per workstation hour (Fig. 6) was reasonably consistent throughout the project. Compared to the relatively large changes in daily productivity (see Fig. 8) when averaged over a month productivity was reasonably constant.

**Figure 7.**
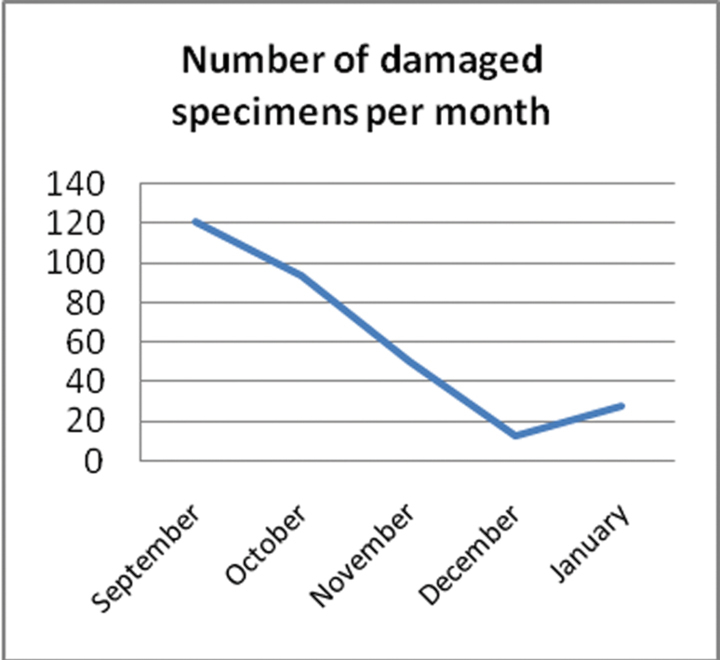
Number of damaged specimens per month.

The number of damaged specimens per month (Fig. 7) was related to the number of specimens being processed and the fragility of the group being worked on. The highest rate of damage was with the Sphingidae moths. All damaged specimens need to be dealt with by collection staff, so an increase in the numbers damaged, meant an increase in collection staff time to remedy.

**Figure 8.**
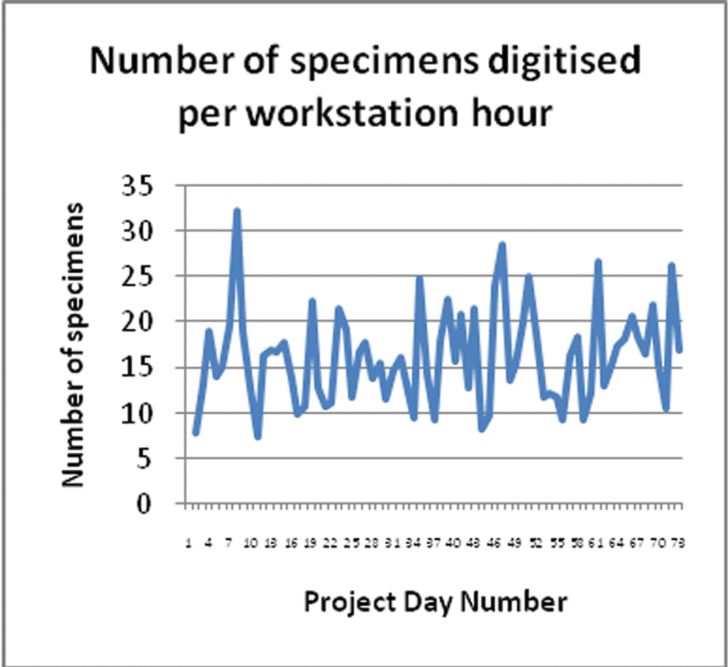
Number of specimens digitised per workstation hour.

**Figure 9.**
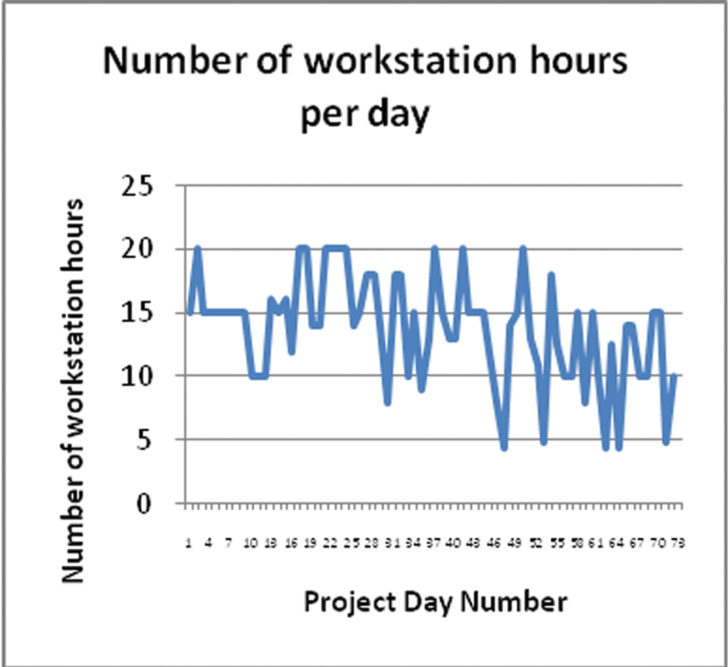
Number of workstation hours per day.

There is great variability in the number of specimens digitised per workstation hour (Fig. 8). This was not simply a factor of the number of workstation hours per day. Other factors, such as volunteer competency and diligence, and the taxonomic group being worked on, also influenced the digitising rate. The variation in the workstation hours (Fig. 9) is due to variable volunteer attendance which was more likely to be affected by external factors than is the case with paid staff, a factor that affects productivity.

## Discussion and conclusions

### Why use image based digitisation?

There was a time when databasing (entry of text only data) of collection holdings was the preferred way of digitising a collection, for collection management and data access. This is now being challenged by the digitising of collections where specimens are imaged and their associated label data entered as complementary data.

The advent of this approach has come from the realisation that having an image of the specimen and its associated labels has strong collection data management benefits including:

• A readily accessible digital voucher of each specimen and its labels for verification and reference as a digital loan

• Reduced need for specimen handling

• A virtual specimen in the event of collection loss or damage, e.g. fire, flood, earthquake, or for when the specimen is on loan

• Remote access to original label data for review by researchers

• A capacity for using handwriting to help identify a collector in the absence of a collector name

• A limited potential for species identification from an image

• Enabling the use of ‘non-experts’ in data entry with the benefit of knowing data quality and enabling dubious data to be checked without having to physically visit a specimen in the collection.

### Best practice

The processes and procedures detailed in this paper constitute best practice for the predefined goal of image based digitising of individual specimens and the associated labels. We have produced documentation and videos that detail the handling of specimens and registers and a handbook for volunteers involved in the digitizing project [Rapid Digitisation Project Resources].

The two most important components of this best practice are:

• the dedicated role of the digitising officer who recruits, trains and coordinates the volunteers, liaises with collection staff and implements the technical processes

• the curation of material prior to digitisation by collection staff which makes the digitising process as effective as possible in terms of consistent identification of digitised specimens, ease of handling and selection of appropriate specimens (including removal of types).

### Curation of specimens by drawer in preparation for digitising

A factor that should not be glossed over is the potential resource impact of preparing drawers of specimens for digitising. This curation involves removing types (where it has been decided that types are not to be imaged by volunteers, as is the case in the AM project), ensuring specimens within the drawers are labelled adequately with taxonomic names and that the specimens are not overcrowded and thus difficult for volunteers to handle. These are tasks which must be carried out by collection staff or, where appropriate, experienced volunteers.

Institutions need to ensure that adequate resources and lead time are made available to allow collection staff to curate drawers well ahead of scheduled digitising for those drawers. In addition, any application for funding of digitising projects needs to factor in resources required for the curation of material to be digitised.

It is difficult to estimate how much time is required specifically for the curation of specimens for digitising (Table 1) because curation is a normal part of collection management. What is clear though is that a dedicated rapid digitising project shifts the priorities of collection staff onto curation of specimens that may otherwise not have been in their workplans. Unless effectively resourced this can lead to conflict over work priorities in the collection. All insect collections contain a mixture of groups which range from well identified (usually because of associated input from skilled researchers) to completely unidentified or incorrectly identified. There may be well-identified groups that are not suitable for digitisation because of their physical state or there may be groups which would be ideal to digitise which are not well identified. The presence of well-identified collection material represents an investment that should not be taken for granted.

### Technical support for creating and maintaining databases and photographic equipment

The entry of metadata for each image captured is an important consideration as it has implications for resourcing, productivity and the ease in which data can be incorporated into the institutions collection management system, in the case of AM that being KE-EMu.

Databasing at the time of image capture could be carried out in a number of ways including: direct entry into a spreadsheet such as Excel, entry into a purpose built database such as MS Access for later/subsequent import/uploading or direct entry into the corporate collection database. We chose the MS Access option because it optimises data entry speed and accuracy (through the use of picklists, default data values and automated field population), and doesn’t carry security overheads (volunteers accessing the corporate database has unacceptable data security issues).

Technical support is required to establish and maintain the database and the various entry forms required.

### Options for Capturing Full Records

The complete label information could be transcribed and entered into a spreadsheet or database at the time of image capture. However, this approach wasn’t adopted because it was felt that separating out the imaging and transcription steps has benefits for the process in terms of specialisation which is likely to result in improvements in speed, efficiency and accuracy.

Imaging the labels allows scope for unlocking and outsourcing the transcription of the complete label data to create a full record.

Two options that utilise volunteers are as follows:

• Internal Volunteers – by setting up separate computer workstations with either spreadsheets or data entry database forms, the label information could be transcribed by the volunteers in the DigiVol laboratory.

• Crowdsourcing with Online Volunteers – the approach chosen was to establish an online volunteer transcription site [Biodiversity Volunteer Portal] where the complete label information can be transcribed into defined database fields. This data can then be validated and imported back into the Emu collection database to create the full database record.

### Funding Options

Our investigation of funding options came to the following conclusion on likely sources of funding for digitising projects:

It is far easier to get funds for buying equipment and building infrastructure than it is for ‘bums on seats’.

With this in mind, some institutions may find it worthwhile to seek funds for equipment purchase and then allocate some existing internal resources to set up the equipment and coordinate the volunteers in a manner that is amenable to their available staff resources.

Short term projects of one or two years may be funded through trusts, particularly those related directly to the institutions activities, e.g. The Australian Museum Foundation. Such short term projects should focus clearly on delivering a specific content such as a charismatic or high profile collection in its entirety.

In the absence of either of these sources of funding it is dependent on the institution itself to determine its priorities in terms of digitisation and focus what resources it can in pursuing those priorities.

### Low cost digitising options

Where institutions are unable to implement best practice because of resourcing constraints the processes and procedures outlined above can be scaled to suit available resources. For example, a single workstation could be established at minimal cost and a small team of volunteers (two to ten) trained and coordinated by an existing staff member if the workstation was located in close proximity to the staff member.

• Equipment selection

The cost of setting up a workstation is somewhat flexible in that many institutions will already have the necessary equipment for specimen handling and curation and also the necessary furniture. This can considerably reduce the costs of setting up a workstation, reducing it to just the cost of imaging equipment and computer software and hardware.

• Computer

A fast but standard specification was chosen to get the best balance between price and performance. Two screens were used: a larger screen for viewing the images (as image capture is controlled through the computer) and another screen for operating the database for data input.

• Copy stands

Good quality copy stands are essential as they provide stability for the camera and a sound platform upon which the specimens can be imaged. Kaiser makes excellent stands.

• Cameras

Any number of cameras could have been chosen and would have been suitable to the task. We chose a Canon 550D as we felt it delivered good results was sufficient to do the job and represented very good value for money. We felt there was no need for a more expensive camera nor a higher resolution camera because we wanted to keep the image size to a manageable 5MB jpeg.

• Storage

When capturing many thousands of images at 5Mb size per image, the impact on storage is significant. Images are stored on the Museums network as part of its image storage infrastructure. Funding for future image capture has been factored into the Museums overall IT planning.
